# Correlation Among Self-Care Ability, Psychological Status, and Quality of Life in Discharged Patients with Hepatolithiasis Complicated with Diabetes Mellitus and T-Tube

**DOI:** 10.3389/fsurg.2022.907900

**Published:** 2022-05-16

**Authors:** Chunqiu Huang, Ying Wang, Yimin Cai, Zhoumin Shen, Honghui Zhang, Zhaoxia Tan, Hongjiao Chen, Bifang Zhou

**Affiliations:** ^1^ Hunan Provincial People’s Hospital (The First-Affiliated Hospital of Hunan Normal University), Changsha, China; ^2^ Department of Nursing, School of Medicine, Hunan Normal University, Changsha, China

**Keywords:** Hepatolithiasis complicated with diabetes mellitus, T-tube, self-care ability, psychological status, quality of life

## Abstract

**Objectives:**

This study aimed to investigate the correlation between self-care ability, psychological status, and quality of life in patients with hepatolithiasis complicated with diabetes mellitus with T-tube.

**Methods:**

The purpose of this study was to select a total of 240 patients with hepatolithiasis complicated with diabetes with T-tube from June to September 2019 in a Third-class Grade A hospital in Changsha, Hunan Province. Self-designed general information questionnaire, self-care ability implementation scale (ESCA), self-rating anxiety scale (SAS), self-rating depression scale (SDS), and quality of life scale (SF-36) were used to conduct a questionnaire survey. The correlation among self-care ability, psychological status, and quality of life of patients with hepatolithiasis complicated with diabetes mellitus with T-tube was analyzed.

**Results:**

The total score of self-care ability of 240 patients with hepatolithiasis combined with diabetes with T-tube was positively correlated with the total score of quality of life (*p* < 0.05). The standard scores of anxiety and depression were negatively correlated with the total score of quality of life (*p* < 0.05). The total score of self-care ability was negatively correlated with the standard score of anxiety and depression (*p* < 0.05).

**Conclusion:**

Improving the self-care ability of patients with hepatolithiasis complicated with diabetes with T-tube and improving their anxiety and depression can improve their quality of life, which provides reference for further study.

## Introduction

Hepatolithiasis, which is defined as the stones formed in each branch bile duct above the confluence of the left and right hepatic ducts, has a high prevalence in Asia (1–3). In western countries, hepatolithiasis has a much lower incidence (4, 5). However, the incidence of hepatolithiasis is increasing in Western countries due to the increase in immigration from high prevalent areas (5–7). Surgical management has long been a primary treatment of hepatolithiasis, and a T-tube is usually retained after operation (8). However, the T-tube placement time is usually 2–3 months, and it can be as long as 3–6 months for complicated cases. Improper nursing will easily lead to complications such as tract infection and T-tube shedding (8).

Diabetes mellitus refers to the presence of hyperglycemia, which is a kind of prevalent chronic lifelong metabolic disease all over the world (9, 10). Various complications caused by long-term metabolic problems and continuous hyperglycemia can gradually damage the patient's tissues and organs, which results in a high mortality rate (11–13). With the change in people's lifestyle and diet structure, the incidence of hepatolithiasis with diabetes is increasing, which seriously affects the quality of life of patients with hepatolithiasis and diabetes with T-tube. Therefore, it is necessary to conduct nursing interventions and take some measures to improve their quality of life.

Previous studies reported a relationship between anxiety and depression, quality of life, and self-care ability of patients with heart failure (14, 15), colorectal cancer (16), or other diseases (17, 18). However, few literature works mention the relationship of these three factors in patients with hepatolithiasis complicated with diabetes mellitus with T tube. The purpose of our study is to research the correlation between self-care ability, psychological status, and quality of life of patients with hepatolithiasis complicated with diabetes mellitus and T-tube to provide a reference for further research.

## Objectives and Methods

### Research Objects

A total of 240 patients with hepatolithiasis complicated with diabetes with T-tube from June to September 2019 in a Third-class Grade A hospital in Changsha, Hunan Province were selected as the research objects.

#### Inclusion and Exclusion Criteria

Inclusion criteria are as follows:

(1) age: ≥18 years and ≤65 years;
(2) medical diagnosis of hepatolithiasis and diabetes mellitus, recovered and discharged with T-tube after surgical treatment; and
(3) informed consent and voluntary participants.

Exclusion criteria are as follows:

(1) those with serious complications or critical illness who can not take care of themselves;
(2) people with a history of mental illness; and
(3) those who have language communication barriers and can not communicate normally.

### Research methods

The main research tools that were used in this study are as follows: a general information questionnaire, self-care ability implementation scale (ESCA), self-rating anxiety scale (SAS), self-rating depression scale (SDS), and quality of life scale (SF-36).

### Survey Tools

#### General Information Questionnaire

The general information questionnaire includes the age, gender, family income, education level, marital status, occupation, main caregivers, and disease-related information of the subjects.

#### Self-Care Ability Implementation Scale

This scale was compiled by American scholars Kearney and Fleischer (19), and Cronbach's *α* coefficient was 0.86–0.92 (20). There are 43 items that include four dimensions: self-concept, self-care responsibility, self-care skills, and health knowledge level. Each item of the scale scored from 0 to 4, with a total score of 172. The higher the score, the stronger the self-care ability. The level of self-care ability is evaluated by a scoring index. The scoring index >66% is in a high grade, 33%–66% is in a medium grade, and <33% is in a low grade.

#### Self-Rating Anxiety Scale

This scale was developed by Zung (21), and Cronbach's *α* coefficient was 0.807. There are 20 items in total, which are described by a standard score. Standard score = total rough score × 1.25. The score index <50 is normal, 50–59 is mild anxiety, 60–69 is moderate anxiety, and >69 is severe anxiety. The higher the standard score, the more severe the symptoms.

#### Self-Rating Depression Scale

SDS was compiled by Zung (22), and Cronbach’s *α* coefficient was 0.854. There are 20 items in total, which are described by a standard score. The higher the standard score, the more severe the symptoms are. The cut-off value of the SDS standard score is 53, of which <53 is normal, 53–62 is mild depression, 63–72 is moderate depression, and >73 is severe depression.

#### Quality of Life Scale (SF-36)

The quality of life scale (SF-36) adopts the Chinese version of the reliability and validity scale tested by Li’s team (23), and Cronbach's *α* coefficient is 0.763. SF-36 has 36 items in total that include eight health dimensions and a self-assessment of health changes. These health dimensions are role-physical (RP), mental health (MH), vitality (VT), bodily pain (BP), social functioning (SF), physiological functioning (PF), general health (GH), and role-emotional (RE). Eight dimensions are divided into the mental health field and the physical health field. The total score of quality of life is the sum of scores in eight dimensions. The higher the score, the better the patient’s quality of life.

### Statistical Methods

Statistical analysis was performed on all experimental data using SPSS 20.0 software. The data on frequency, percentage, and Spearman rank correlation were analyzed.

## Results and Discussion

In this study, a total of 248 questionnaires were sent out, of which 240 were valid. The effective recovery rate was 96.7%. There are 87 males and 153 females (Table 1).

**Table 1:** General information of research objects (*n *= 240, %).

Variable	Category	Frequency	Percentage
Gender	Man	87	36.25
	Woman	153	63.75
Age	18–30 years	29	12.08
	31–45 years	181	75.42
	>45 years	30	12.50
Marital status	Unmarried	55	22.92
	Married	185	77.08
Educational level	Junior high school or below	135	56.25
	High school	74	30.83
	Junior college degree or above	31	12.92
Occupation	Farmer	103	42.92
	Enterprise and public institution	63	26.25
	Others	74	30.83
Monthly household income	≤2,000 RMB	146	60.83
	2,001–5,000 RMB	74	30.83
	≥5,000 RMB	20	8.33
First operation	Yes	133	55.42
	No	107	44.58
Days after operation	≤14 days	144	60.00
	15–30 days	62	25.83
	>30 days	34	14.17
Sequence of diagnosis of hepatolithiasis and diabetes mellitus	Hepatolithiasis first	203	84.58
	Diabetes mellitus first	18	7.50
	Both at the same time	19	7.92
Diagnosis time of diabetes mellitus	<6 months	89	37.08
	6 months–3 years	104	43.33
	>3 years	47	19.58
First discharge with T-tube	Yes	219	91.25
	No	21	8.75
T-tube clamped	Yes	198	82.50
	No	42	17.50
Recovery status of wound	Well	216	90.00
	Worse	24	10.00
Main caregiver	By other people	180	75.00
	By myself	60	25.00
Nursing ways of T-tube	By medical institution	69	28.75
	Nurse’s home care	116	48.33
	Self-care	55	22.92
Control of blood sugar	Good	65	27.08
	Bad	93	38.75
	Not monitored	82	34.17

As shown in Table 2, the total score of self-care ability was negatively correlated with the standard score of anxiety and depression (*r *= −0.424, −0.400, *p* < 0.05). Additionally, the score of each dimension has negative significance with psychological status. Therefore, the stronger the self-care ability of patients with hepatolithiasis and diabetes with T tube, the better their psychological status, and vice versa. This is consistent with the research results of scholars such as Patrick (24). The reason may be that patients with good self-care ability know the importance of self-care, which can stimulate their self-responsibility to actively learn relevant health knowledge and nursing skills from passive acceptance to active participation. Patients know more about diseases and nursing knowledge so that they can treat diseases correctly and maintain a good psychological state to enhance patients’ confidence in overcoming diseases.

**Table 2:** Correlation between self-care ability and psychological status of discharged patients with hepatolithiasis and diabetes mellitus with T-tube (*n* = 240).

Variable	Statistics	Standard score of anxiety	Standard score of depression
Total score of self-care ability	*r*	−0.424**	−0.400**
	*p*	<0.001	<0.001
Self-concept	*r*	−0.508**	−0.353**
	*p*	<0.001	<0.001
Self-care responsibility	*r*	−0.320**	−0.234**
	*p*	<0.001	<0.001
Self-care skills	*r*	−0.144*	−0.192**
	*p*	0.026	0.003
Health knowledge level	*r*	−0.201**	−0.296**
	*p*	0.002	<0.001

*p < 0.05; **p < 0.01.

The results of Table 3 showed that the total score of the SF-36 scale was positively correlated with the total score of self-concept, self-care responsibility, self-care skills, and health knowledge level (*r *= 0.263, *p *< 0.05). It is consistent with the results of Patrick (24) and Kessing (25). From the table, we found that the higher quality of life of patients with hepatolithiasis complicated with diabetes mellitus and T-tube was closely related to their stronger self-care ability. The main reasons are that patients having strong self-concept and awareness of self-recognition can cooperate with the medical treatment positively so as to improve their body function and their quality of life. Therefore, nursing staff should improve patients’ weaknesses in personalize training and health education in continuing home care to improve their quality of life.

**Table 3:** Correlation between self-care ability and quality of life in discharged patients with hepatolithiasis and diabetes mellitus with T-tube (*n* = 240).

Variable	Statistics	Total score of self-care ability	Self-concept	Self-care responsibility	Self-care skills	Health knowledge level
Total score of SF-36	*r*	0.263**	0.242**	0.231**	0.239**	0.108
	*p*	<0.001	<0.001	<0.001	<0.001	0.094
Physiological functioning (PF)	*r*	0.235**	0.184**	0.234**	0.230**	0.123
	*p*	<0.001	0.004	<0.001	<0.001	0.058
Role-physical (RP)	*r*	0.136*	0.101	0.166**	0.103	0.035
	*p*	0.036	0.120	0.010	0.112	0.594
Bodily pain (BP)	*r*	0.037	0.164*	0.111	0.051	−0.109
	*p*	0.572	0.011	0.086	0.434	0.091
General health (GH)	*r*	−0.089	−0.004	−0.071	−0.155*	−0.034
	*p*	0.170	0.952	0.271	0.016	0.603
Vitality (VT)	*r*	0.069	0.068	0.078	−0.015	0.079
	*p*	0.287	0.295	0.231	0.812	0.224
Social functioning (SF)	*r*	0.189**	0.143*	0.127*	0.175**	0.132*
	*p*	0.003	0.026	0.049	0.007	0.041
Role-emotional (RE)	*r*	0.204**	0.169**	0.154*	0.272**	0.043
	*p*	0.001	0.009	0.017	<0.001	0.509
Mental health (MH)	*r*	0.047	0.194**	−0.007	−0.015	0.011
	*p*	0.473	0.003	0.908	0.818	0.863

*p < 0.05; **p < 0.01.

As shown in Table 4, the total score of the SF-36 scale was negatively correlated with the standard score of anxiety and depression (*r *= −0.285, −0.266, *p *< 0.05). Therefore, the better the psychological status of discharged patients with hepatolithiasis and diabetes mellitus with T-tube, the higher the patients’ quality of life, and vice versa. The results were consistent with the research results of Patrick (24) and Park (26). Although hepatolithiasis is a benign disease, it has a high recurrence rate with some complications (27). Diabetes mellitus is a typical physical and mental disease with a long course and many complications (28). Patients with hepatolithiasis complicated with diabetes mellitus and T-tube are prone to psychological problems and stress due to their awareness of the difficulty in curing their diseases completely and the inconvenience of these diseases to their daily life and social work, which will lead to abnormal emotional control and result in a significant negative impact on their quality of life. Therefore, in the management and treatment of patients with hepatolithiasis complicated with diabetes, we should not only pay attention to the improvement of patients’ physiological functions but also intervene in the factors that affect their psychology and life behavior.

**Table 4:** Correlation between psychological status and quality of life in discharged patients with hepatolithiasis and diabetes mellitus with T-tube (*n* = 240).

Variable	Statistics	Standard score of anxiety	Standard score of depression
Total score of SF-36	*r*	−0.285**	−0.266**
	*p*	<0.001	<0.001
Physiological functioning (PF)	*r*	−0.362**	−0.371**
	*p*	<0.001	<0.001
Role-physical (RP)	*r*	−0.051	−0.114
	*p*	0.428	0.078
Bodily pain (BP)	*r*	−0.011	0.062
	*p*	0.868	0.337
General health (GH)	*r*	0.086	0.079
	*p*	0.185	0.224
Vitality (VT)	*r*	−0.170**	−0.087
	*p*	0.008	0.177
Social functioning (SF)	*r*	−0.346**	−0.326**
	*p*	<0.001	<0.001
Role-emotional (RE)	*r*	−0.079	−0.077
	*p*	0.224	0.237
Mental health (MH)	*r*	−0.356**	−0.244**
	*p*	<0.001	<0.001

*p < 0.05; **p < 0.01.

## Conclusion

Our study focused on exploring the correlation between self-care ability, psychological status, and quality of life in discharged patients with hepatolithiasis complicated with diabetes mellitus and T-tube. Data showed that (1) patients’ self-care ability was negatively associated with their psychological status (*p* < 0.05), (2) self-care ability of 240 patients with hepatolithiasis combined with diabetes and T-tube was positively correlated with their quality of life (*p* < 0.05), and (3) psychological status has a negative difference with the quality of life (*p* < 0.05). From the results, it can be seen that stronger self-care ability and lower anxiety and depression level were closely related to the higher quality of life.

In this work, we demonstrated that promoting the self-care ability of patients with hepatolithiasis complicated with diabetes and T-tube and improving their psychological status can improve their quality of life. Our findings highlight the necessity of psychological interventions and guidance of self-recognition of patients, which provide a reference for further study on the improvement of quality of life for this population.

## Data Availability

The original contributions presented in the study are included in the article/Supplementary Material; further inquiries can be directed to the corresponding author/s.
